# Peripheral immune-based biomarkers in cancer immunotherapy: can we realize their predictive potential?

**DOI:** 10.1186/s40425-019-0799-2

**Published:** 2019-11-27

**Authors:** Andrew B. Nixon, Kurt A. Schalper, Ira Jacobs, Shobha Potluri, I-Ming Wang, Catherine Fleener

**Affiliations:** 1 Duke University School of Medicine, Department of Medicine/Medical Oncology, 133 Jones Building, Research Drive, Durham, NC 27710 USA; 2 Yale School of Medicine, Translational Immuno-Oncology Laboratory, Yale Cancer Center, 333 Cedar St. FMP117, New Haven, CT 06520-8023 USA; 3 Pfizer Inc, Early Oncology Development and Clinical Research, 219 East 42nd St, New York, NY 10017-5755 USA; 4 Pfizer Inc., Computational Biology, 230 E Grand Ave, South San Francisco, CA 94080 USA; 5 Pfizer Inc., 10777 Science Center Dr., San Diego, CA 92121 USA; 6 Pfizer Inc., Translational Oncology, La Jolla, CA USA; 7 Present Address: Translational Science at Samumed, LLC, La Jolla, CA USA

**Keywords:** Biomarkers, Immunology, Immunotherapy, Oncology, Peripheral blood

## Abstract

The immunologic landscape of the host and tumor play key roles in determining how patients will benefit from immunotherapy, and a better understanding of these factors could help inform how well a tumor responds to treatment. Recent advances in immunotherapy and in our understanding of the immune system have revolutionized the treatment landscape for many advanced cancers. Notably, the use of immune checkpoint inhibitors has demonstrated durable responses in various malignancies. However, the response to such treatments is variable and currently unpredictable, the availability of predictive biomarkers is limited, and a substantial proportion of patients do not respond to immune checkpoint therapy. Identification and investigation of potential biomarkers that may predict sensitivity to immunotherapy is an area of active research. It is envisaged that a deeper understanding of immunity will aid in harnessing the full potential of immunotherapy, and allow appropriate patients to receive the most appropriate treatments. In addition to the identification of new biomarkers, the platforms and assays required to accurately and reproducibly measure biomarkers play a key role in ensuring consistency of measurement both within and between patients. In this review we discuss the current knowledge in the area of peripheral immune-based biomarkers, drawing information from the results of recent clinical studies of a number of different immunotherapy modalities in the treatment of cancer, including checkpoint inhibitors, bispecific antibodies, chimeric antigen receptor T cells, and anti-cancer vaccines. We also discuss the various technologies and approaches used in detecting and measuring circulatory biomarkers and the ongoing need for harmonization.

## Introduction

Immunotherapy represents a major breakthrough for a number of cancers, but not all patients derive benefit, leaving many with an unmet need. When considering the immune composition of the tumor, factors such as the amount, functionality, and spatial organization of infiltrated immune cells, particularly T cells [1], are established as important for immune checkpoint therapy responses, for example. Other tumor factors associated with enhanced response to immunotherapy include microsatellite instability, tumor mutational burden (TMB) [2–4], and inflammatory gene expression [5]. Recently, the analysis of TMB and T-cell gene expression provided value in identifying patients most likely to respond to pembrolizumab, suggesting the potential value for these biomarkers in the selection of patients for checkpoint therapy [5].

While tumor sampling is widely implemented for biomarker identification and analysis, obtaining tissue is challenging because of limited accessibility, multiple lesions, heterogeneity of the biopsy site, and patient condition. Tumor biopsies are generally costly, invasive, cause treatment delays, and increase the risk of adverse events (AEs). Hence, analysis of readily accessible peripheral blood is critical for developing biomarkers with clinical utility. Tumor genomic alterations such as discrete oncogenic variants (e.g. *EGFR, PBRM1*, *LKB1*, *JAK1/2*, and *B2M* mutations), complex rearrangements/copy number variations (e.g. programmed death ligand 1/2 [PD-L1/2] amplification), microsatellite instability, and TMB-related metrics can be detected in blood using next-generation sequencing (NGS) analysis of circulating tumor DNA. Circulating tumor cells also demonstrate prognostic value as liquid biopsies in certain tumor types such as breast and prostate, with measurement of nuclear proteins such as prostate cancer androgen receptor splice variant-7, providing additional supportive information for prognosis and therapy selection [6]. For evaluation of peripheral immune-cell function, several immune-related analytes may be measured, including cytokines, soluble plasma proteins, and immune cells, analyzed by surface marker expression, transcriptomic, or epigenetic profiles. Table 1 lists example technologies that may be employed for the measurement of circulating biomarkers. Of these, RNA-seq, flow and mass cytometry, and enzyme-linked immunosorbent assay-based multiplex technologies are frequently utilized to identify peripheral immune markers associated with clinical response to immune modulating therapies.

**Table 1:** Approaches for measuring peripheral biomarkers

Approach	Sample	Strengths	Manufacturers and/or examples of technologies
Whole transcriptome profiling, RNA- seq, single-cell RNA-seq	RNA from PBMCs	RNA-seq • Fast and high efficiency • Broad, dynamic range • Detects differentially expressed genes • Measures average expression level • Uses millions of short reads (sequence strings), so all RNA in a sample can be investigated	• Illumina
		Single-cell (scRNA-seq) • Measures the distribution of expression levels for each gene • Expression patterns of individual cells can be defined in complex tissues • High resolution of cell-to-cell variation	• Bio-Rad® single-cell RNA-sequencing solution • 10X Genomics
Epigenetic differentiation-based immune-cell quantification	Genomic DNA from fresh or frozen whole blood, PBMCs	• Broad range of acceptable sample conditions (e.g. samples can be frozen and shipped without other steps) • Standardized measurements and circulating and tissue-infiltrating immune cells can be compared as an alternative to flow cytometry for peripheral blood samples and IHC for solid tissues	• Quantitative real-time PCR-assisted cell counting
Chromosomal confirmation signatures	Blood	• CCSs can provide a stable framework from which changes in the regulation of a genome can be analyzed	• EpiSwitch™
Protein microarray	Fresh or frozen serum and plasma	• Versatile and robust platform • Miniaturized features, high throughput, and sensitive detections • Reduction in sample volume used • Variety of biological samples can be analyzed	• ProtoArray® (Life Technologies) can analyze serologic response of 9000 proteins simultaneously • SOMAscan® Assay • Olink Proteomics
Mass spectrometry	Blood	• Mass spectrometry-based protein measurements in blood	• Biodesix
Flow cytometry	Blood, fresh or frozen PBMCs, circulating tumor cells	• Multiparameter measurements at single-cell level • Rapid, high throughput manner • Cytometers available at reasonable cost • Recent advances in lasers/fluorochrome technology allows multiparameter analysis of rare cells (e.g. tumor antigen-specific T lymphocytes)	• BD LSRFortessa™ X-20 • BD FACSymphony™
Mass cytometry	Blood, fresh or frozen PBMCs	• Multiparameter single-cell analysis • Heavy metal ions as antibody labels overcome limitations of fluorescence-based flow cytometry • Little overlap between channels and no background (up to 40 labels per sample) • Increased number of phenotypic and functional markers can be probed	• Comprehensive analysis of profile and function of immune populations (e.g. time-of-flight cytometry by Helios™)
T- and B-cell receptor deep sequencing	PBMCs formalin-fixed paraffin-embedded	• Identify changes in T- & B-cell populations, both in circulation and within tumors • Millions of T- & B-cell receptor sequences can be read from a single sample • Identify clonal expansion (measure of adaptive immune response) • Has been used to show clinical response to cancer immunotherapy	• ImmunoSEQ® immune profiling system at the deep level (Adaptive Biotechnologies) • Illumina HiSeq system
ELISA and multiplex assays	Blood	• Widely used • Multiple biomarkers measured at once • Small sample volume • Measures soluble mediators (e.g. cytokines, chemokines, autoantibodies)	• ELISA • Multianalyte immunoassays: Simple Plex™ MesoScaleDiscovery Luminex, Quanterix™

*CCS* Chromosomal confirmation signature, *ELISA* Enzyme-linked immunosorbent assay, *IHC* Immunohistochemistry, *PBMC* Peripheral blood mononuclear cell, *PCR* Polymerase chain reaction

Many studies provide compelling evidence that peripheral immune fitness and status may aid in guiding treatment decisions. Thus far, no US FDA-approved circulatory immunological biomarker has been validated for patients with cancer, and significant challenges exist in bridging the gap between identifying signatures correlated with response, and validated prospective and predictive biomarker selection. As the importance of biomarkers to guide therapies escalates, the need for proper analytical and clinical validation for these biomarkers is paramount. Analytical validation ensures the biomarker technically performs for the intended purpose and has reproducible performance characteristics. Once analytically validated, it can then be evaluated for clinical utility where iterative testing can link the biomarker to a biological process or clinical outcome. In order to adopt biomarkers more quickly and effectively, this increased emphasis on analytical and clinical validation is paramount. In terms of approaching biomarker development for peripheral cell analyses, pre-analytical considerations around collection methodology, vacutainer type, processing time, and storage conditions are key. Furthermore, differences in technologies, antibodies, and development of multiplex panels may lead to variability within these molecular correlates.

This review focuses on key findings correlating peripheral blood immune biomarkers at baseline or on treatment with response to immunotherapies of various modalities, their associated methodologies, and emerging technologies showing promise for deeper profiling and insights.

## Biomarkers and immunotherapy modalities

### Peripheral immune-based biomarkers

**Fig. 1:**
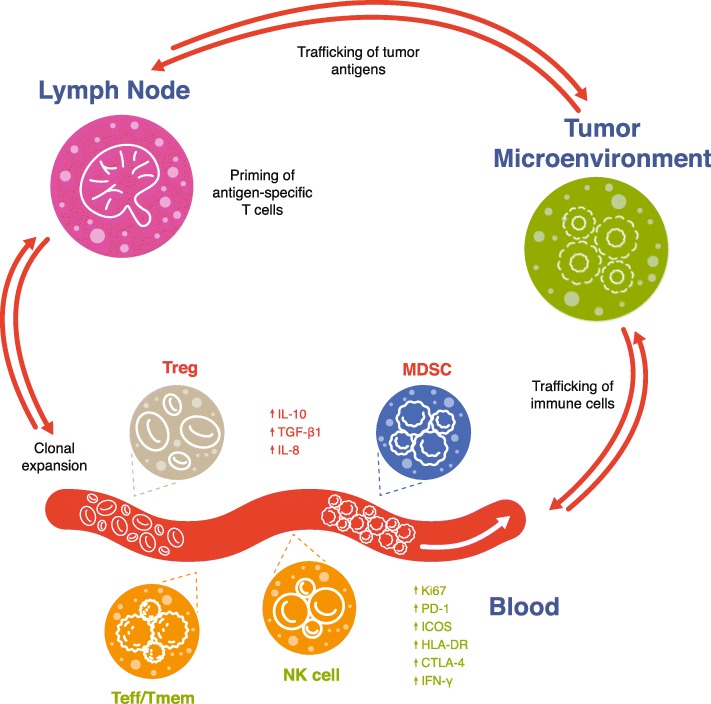
Representation of key peripheral immune cells associated with clinical response to immunotherapy. Green text represents cells and markers associated with better response to immunotherapy, while red text designates cells associated with poorer immunotherapy response. *MDSC,* myeloid-derived suppressor cell; *NK,* natural killer; *Teff*, effector T cell; *Tmem* memory T cell; *Treg,* regulatory T cell.

Some important peripheral leukocyte subtypes demonstrating associations with responses to immunotherapy are shown in Fig. 1. Baseline or on-treatment frequencies of effector cells are often associated with positive treatment outcomes, while high frequencies of inhibitory cells such as myeloid-derived suppressor cells (MDSCs) and regulatory T cells (Treg) often associate with poorer response. The specific cell types and kinetics of cell responses are inconsistent across studies, which may reflect differences in methodologies, sample matrix or assay reagents used, validation rigor, patient tumor stage, or prior and current treatments. Table 2 summarizes some key findings of reviewed literature regarding the current landscape of predictive immune-based circulating biomarkers across immunotherapy treatment modalities.

**Table 2:** Immunotherapy modalities and key peripheral findings associated with response

Indication	Modality	Treatment	Number of patients	Peripheral finding associated with clinical response	Reference
Melanoma	ICI	Anti-PD-1	40	Higher baseline frequency of Bim^+^PD-1^+^CD8 T cells in responders. Levels of Bim decreased after 3 months of treatment	[7] Dronca 2015
Melanoma	ICI	Ipilimumab	137	Higher frequency of baseline CD8 EM1, trend for lower TEMRA, and on treatment decreases in PD-1 associated with improved BOR and OS	[8] Wistuba-Hamprecht 2017
Metastatic melanoma	ICI	Ipilumumab/pembrolizumab	30	Low baseline CD45RO^+^ CD8^+^ associated with non-response and poorer OS for ipilimumab, but not pembrolizumab	[9] Tietze 2017
Stage IV melanoma	ICI	Pembrolizumab/prior ipilimumab	29	Clinical outcome related to the ratio of T_ex_-cell reinvigoration to tumor burden. Patients with longer PFS had low tumor burden and clustered above the fold-change of T_ex-_cell reinvigoration to tumor-burden regression line. Findings supported by independent validation cohort	[3] Huang 2017
Metastatic melanoma	ICI	Ipilimumab/nivolumab	190	Low PD-L1 on CD4/8^+^ T cells prognostic for greater OS/PFS; CD137^+^ CD8 T cells predicted lack of relapse to ipilimumab + nivolumab combination	[10] Jacquelot 2017
Metastatic melanoma	ICI	Anti-PD-1	30	Increased baseline HLA-DR, CLTA-4, CD56, and CD45RO associated with response; elevated CD14^+^CD16b^−^HLA^−^DR^hi^ identified as potential predictor of response. Findings supported by independent validation cohort	[11] Krieg 2018
Melanoma	ICI	Ipilimumab and anti-PD-1	67	For ipilimumab, lower levels of baseline memory (CD45RA^+^) T cells associated with response; for anti-PD-1, increased CD69^+^ NK cells in PMA/ionomycin stimulated PBMCs in responders	[12] Subrahmanyam 2018
Stage IV melanoma	ICI	Ipilimumab and local radiotherapy	22	Higher baseline CD8 CM cells, transient on-treatment increases in MIP-1α and β, and sustained increases in IP-10 and MIG associated with CR/PR	[13] Hiniker 2016
Melanoma	ICI	Ipilimumab, anti-PD-1 or combination	39	Increases in CD21^lo^ B cells and in plasmablasts after combination therapy associated with incidence of IRAEs	[14] Das 2018
Melanoma	ICI	Ipilimumab	83	Higher baseline monocytic MDSC associated with shorter OS	[15] Kitano 2014
Melanoma	ICI	Ipilimumab	49	Lower frequency of monocytic MDSC associated with clinical response	[16] Meyer 2014
Advanced melanoma	ICI	Neoadjuvant ipilimumab	35	On treatment decrease in MDSC and increase in Treg associated with improved PFS	[17] Tarhini 2014
Metastatic melanoma NSCLC	ICI	Nivolumab, pembrolizumab; nivolumab/ipilimumab combination	29	On-treatment decreases in serum IL-8 between baseline and best response, which increased on progression	[18] Sanmamed 2017
Stage 1B-IIIA NSCLC	ICI	Ipilimumab, neoadjuvant chemotherapy, paclitaxel	24	Increased T cell ICOS, HLA-DR, CTLA-4, and PD-1 after ipilimumab, but no association with response	[19] Yi 2017
Urothelial	ICI	Ipilimumab	6	Increased on-treatment ICOS^+^ CD4^+^ and NY-ESO-1 responsive T cells (correlation with clinical outcome not reported)	[20] Liakou 2008
ER^+^/PR^+^ breast cancer	ICI	Tremelimumab and exemestane	26	Compared with PD, patients with SD had greater increase in ICOS on T cells and an increase in the ratio of ICOS^+^ T cells to Treg in blood	[21] Vonderheide 2010
NSCLC, Melanoma	ICI	Nivolumab	83	Longer PFS in patients with high T cell CM/effector ratio associated with inflammatory gene transcripts in tumor at baseline	[22] Manjarrez-Orduno 2018
Advanced NSCLC	ICI	Pembrolizumab, nivolumab, or atezolizumab	29	Early on-treatment proliferative responses in PD-1^+^ CD8^+^ T cells associated with PR or SD	[23] Kamphorst 2017
Various	ICI	Pembrolizumab or nivolumab	25	On treatment increases in PD-1 on CD4^+^ and NK cells in responders; decreases in GITR^+^ on NK cells, CD4^+^, CD8^+^ T cells; decreases in CTLA-4 on NK cells and OX40 on CD4^+^ T cells	[24] Du 2018
Ovarian, gastric cancer ascites	Bispecific Ab	Catumaxomab (EpCAM/CD3 bispecific)	258	Higher relative lymphocyte count pre-treatment associated with longer OS. On-treatment HAMA associated with greater puncture-free survival, OS, and time to next therapeutic paracentesis	[25] Heiss 2014
ALL	Bispecific Ab	Blinatumomab (CD19 BiTE)	42	High baseline Treg predictive of non-response	[26] Duell 2017
Melanoma	Cancer vaccine	Multi-epitope peptide vaccine	37	Ability of CD8^+^ T cells to produce IFN-γ after ex vivo stimulation with the vaccinating melanoma peptides correlated with clinical responses to the vaccine	[27] Schaefer 2015
mCRPC	Cancer vaccine	DCvac and docetaxel	43	On-treatment decreases in peripheral MDSCs were associated with improved survival	[28] Kongsted 2017
CRPC	Cancer vaccine	DNA vaccine encoding prostatic acid phosphatase	38	Non-immune responder patients tended to have higher antigen-specific IL-10 secretion prior to vaccination	[29] Johnson 2017
CRPC	Cancer vaccine	Personalized peptide vaccine	40	4-gene classifier (*LRRN3, PCDH17, HIST1H4C*, and *PGLYRP1)* and elevated baseline IL-6 associated with shorter survival	[30] Komatsu 2012
mCRPC	Cancer vaccine	PROSTVAC and ipilimumab	30	Lower baseline PD-1^+^Tim-3^NEG^ CD4_EM_, and higher baseline PD-1^NEG^TIM-3^+^CD8 and CTLA4^NEG^ Treg associated with improved OS. An increase in Tim-3^+^ NK cells post- vs. pre-vaccination associated with longer OS	[31] Jochems 2014
CRPC	Cancer vaccine	Prostate GVAX and ipilimumab	28	Baseline elevated CD4^+^CTLA-4^+^ predicted survival. High pre-treatment levels of CD14^+^HLA-DR^─^ monocytic MDSC were associated with reduced OS	[32, 33] Santegoets 2013, 2014
Advanced NSCLC	Cancer vaccine	TG4010 and gemcitabine/cisplatin	148	Normal baseline levels of CD16^+^CD56^+^CD69^+^ lymphocytes associated with better clinical outcome compared with chemotherapy alone	[34] Quoix 2011
NSCLC	Cancer vaccine	RNActive®CV9201	22	On-treatment transcriptional modules associated with T and NK cells correlated with prolonged PFS; confirmed correlation by flow cytometry	[35] Hong 2016
Pancreatic cancer	Cancer vaccine	3 therapeutic epitope peptides and gemcitabine	63	Lower PD-1^+^ CD4 and 8 T cells and Tim-3^+^CD8^+^ T cells associated with longer survival	[36] Shindo 2017
MUC1^+^ advanced / recurrent NSCLC	Cancer vaccine	MUC1 peptide loaded dendritic cell-based vaccine	40	irAEs and higher baseline lymphocyte count were predictive of response	[37] Teramoto 2017
CLL	CAR-T	CTL019	41	Peripheral expansion of T cells in CTL019 product associated with response; elevated on treatment IL-15, IL-7, and IL-6 in CR and a subset of PR	[38] Fraietta 2018
DLBCL, MCL, ALL, FL, CLL	CAR-T	Autologous CD19 CAR-T	15	Baseline Th1 immune fitness, low monocytic MDSC correlated with response; high baseline or increasing on-treatment monocytic MDSC, high IL-6, IL-8, NAP-3, PD-L1, and PD-L2 correlated with poorer survival	[39] Enblad 2018
DLBCL, PMBCL, TFL	CAR-T	Axicabtagene ciloleucel	111	CAR-T expansion (higher AUC to day 28) correlated with response. Elevated serum IL-6, IL-10, IL-15, IL-2Rα associated with neurological events and CRS	[40] Neelapu 2017
Relapsed or refractory CD19^+^ B-ALL	CAR-T	CD19 CAR-T with defined CD4/8 ratio	29	Loss of CD19 target antigen or development of CD8^+^ immunity to CAR product associated with relapse	[41] Turtle 2016
mCRC	CAR-T	Anti-CEA CAR-T	6	Increases in NLR and serum IL-6 positively correlated with response; lower NLR fold-change correlated with serological decreases in CEA	[42] Saied 2014
DLBCL, FL, MCL	CAR-T	Autologous CD19 CAR-T	22	Pre-infusion polyfunctional T cells in drug product, CAR-T expansion, and baseline serum IL-15 associated with response. Antitumor efficacy associated with polyfunctional IL-17A producing T cells	[43] Rossi 2018

*Ab* Antibody, *ALL* Acute lymphoblastic leukemia, *AUC* Area under the curve, *BiTE* Bispecific T-cell engager, *BOR* Best overall response, *CAR* Chimeric antigen receptor, *CAR T* CAR T cell, *CC* Cholangiocarcinoma, *CEA* Carcinoembryonic antigen, *CLL* Chronic lymphocytic leukemia, *CM* Central memory, *CPRC* Castrate-resistant prostate cancer, *CR* Complete response, *CRS* Cytokine-release syndrome, *CTLA-4* Cytotoxic T-lymphocyte-associated protein 4, *DCvac* Dendritic cell vaccination, *DLBCL* Diffuse large B-cell lymphoma, *EM* Effector memory, *ER* Estrogen receptor, *FL* Follicular lymphoma, *GC* Gastric cancer, *GITR* Glucocorticoid-induced TNFR-related protein, *HAMA* Human anti-mouse antibody, *ICI* Immune checkpoint inhibitor, *IL* Interleukin, *irAE* Immune-related adverse event, *MCL* Mantle cell lymphoma, *mCRC* Metastatic colorectal cancer, *mCRPC* Metastatic castration-resistant prostate cancer, *MDSC* Myeloid-derived suppressor cell, *MIG* Monokine induced by interferon-gamma, *MIP* Macrophage inflammatory protein, *MUC1* Mucin 1, *N/A* Not applicable, *NK* Natural killer, *NLR* Neutrophil-to-lymphocyte ratio, *NSCLC* Non-small cell lung carcinoma, *OS* Overall survival, *PBMC* Peripheral blood mononuclear cell, *PD* Progressive disease, *PD-1* Programmed cell death 1, *PD-L1* Programmed death ligand 1, *PD-L2* programmed death ligand 2, *PFS* Progression-free survival, *PMBCL* Primary mediastinal large B-cell lymphoma, *PR* Partial response, *PR+* Progesterone receptor positive, *RCC* Renal cell carcinoma, *SCLC* Small cell lung cancer, *SD* Stable disease, *TEMRA* Terminally differentiated effector-memory T cells, *TFL* Transformed follicular lymphoma, *TNFR* Tumor necrosis factor receptor, *Treg* Regulatory T cell

### Checkpoint inhibitors

Activated, exhausted, and target-bearing lymphocytes can be assessed through multiparameter immunophenotypic analysis to facilitate patient stratification. Changes in biomarkers following initial treatment could also potentially screen for early response. For example, in patients with advanced cancer, responders showed a higher expression of programmed cell death protein 1 (PD-1) on CD4^+^ and natural killer (NK) cells than non-responders after the first cycle of anti-PD-1 immunotherapy, with lower expression of T-cell CTLA-4, glucocorticoid-induced TNFR-related protein, and OX40 after the second cycle. The elevation of key immune metrics following the first cycle, with a decrease after the second, was associated with a better outcome at an early treatment stage [24]. Tumor burden has been shown to correlate with PD-1 expression on peripheral lymphocytes, and PD-1 engagement in vivo can be measured on circulating T cells as a biomarker for response to immunotherapy [7, 44]. Immune metrics currently associated with sensitivity/resistance to PD-1 blockers include early changes in peripheral T-cell proliferation [3] and serum levels of interleukin 8 (IL-8) [18]. Notably, a surrogate marker of blood TMB has been shown to identify patients with improvements in progression-free survival (PFS) after treatment with the anti-PD-L1 antibody atezolizumab [45].

### Melanoma

In some studies of checkpoint inhibitors, the assessment of blood before and on-treatment has provided insights into patients’ immune characteristics and how these relate to response to therapy. An analysis of peripheral blood mononuclear cells (PBMCs) before and during ipilimumab treatment in 137 late-stage melanoma patients found memory and baseline-naïve T cells correlated with overall survival (OS) [8]. Baseline CD8 effector-memory type 1 (EM1) cells positively associated with OS, whereas terminally differentiated effector-memory CD8 cells (TEMRA CD8) negatively associated with OS [8], suggesting CD8 EM1 cells may predict the clinical response to ipilimumab.

During a prospective assessment of clinical data from 30 patients with melanoma prior to anti-CTLA-4 treatment (ipilimumab, *n* = 21) or anti-PD-1 treatment (pembrolizumab, *n* = 9), baseline CD45RO^+^CD8^+^ T-cell levels correlated with ipilimumab response. Patients with normal baseline levels of CD45RO^+^CD8^+^ T cells had significantly longer OS with ipilimumab but not pembrolizumab treatment, and the activation of CD8^+^ T cells appeared to be non-antigen-specific. The authors concluded that baseline levels of CD45RO^+^CD8^+^ T cells constitute a promising biomarker for predicting the response to ipilimumab [9].

T-cell reinvigoration and immune contexture before and after treatment may be assessed with RNA sequencing and whole exome sequencing. Recently the peripheral blood of 29 patients with stage IV melanoma was profiled using flow and mass cytometry, along with RNA sequencing before and after pembrolizumab treatment to identify altered pharmacodynamics of circulating exhausted-phenotype CD8 T (T_ex_) cells [3]. Immunologic responses were seen in most patients; however, imbalances between tumor burden and T-cell reinvigoration were associated with a lack of benefit. Patients with longer PFS had a low tumor burden and banded above the fold-change of T_ex_-cell reinvigoration to tumor-burden regression line, implying clinical outcome was related to the ratio of T_ex_-cell reinvigoration to tumor burden [3]. An independent cohort of patients with advanced melanoma treated with pembrolizumab was analyzed by flow cytometry, supporting the relationship between reinvigorated CD8 T cells in the blood and tumor burden, and the correlation with clinical outcome. Interestingly, in an analysis of eight pooled cohorts including baseline samples from 190 patients with unresectable melanoma, elevated PD-L1 expression on peripheral blood CD4^+^ and CD8^+^ T cells predicted resistance to CTLA-4 blockade. Moreover, in resected stage III melanoma cells, detectable CD137^+^CD8^+^ peripheral blood T cells predicted lack of relapse with ipilimumab plus nivolumab [10]. Expression of PD-L1 on blood CD8^+^ T cells could be a valuable marker of sensitivity to CTLA-4 inhibition [10].

In a recent study using a bioinformatics pipeline and high-dimensional, single-cell mass cytometry, the immune-cell subsets before and after 12 weeks of anti-PD-1 immunotherapy were analyzed in 20 patients with stage IV melanoma [11]. During treatment there was a response to immunotherapy in the T-cell compartment in peripheral blood. Before therapy, however, the frequency of CD14^+^CD16 − HLA-DR^hi^ monocytes predicted response to anti-PD-1 immunotherapy. The authors confirmed their results in an independent validation cohort using conventional flow cytometry, concluding that the frequency of monocytes in PBMCs may support clinical decisions [11].

In another study employing mass cytometry, the peripheral blood of patients with melanoma was profiled to find predictive biomarkers of response to anti-PD-1 or anti-CTLA-4 therapy [12]. Analysis of samples from 67 patients using approximately 40 surface and intracellular markers indicated distinct predictive biomarker candidates for anti-CTLA-4 and anti-PD-1 immunotherapy. CD4^+^ and CD8^+^ memory T-cell subsets were cited as potential biomarker candidates for anti-CTLA-4 response, whereas, for anti-PD-1 therapy, NK-cell subsets (MIP-1β- and CD69-expressing NK cells) were increased in patients with clinical responses [12]. The findings are validated to some extent in a separate study, wherein memory subsets were predictive of response to CTLA-4 blockade in patients with melanoma [13].

Using flow and mass cytometry, combined checkpoint inhibition was studied in patients with advanced melanoma compared with patients receiving either anti-CTLA-4 or anti-PD-1 alone [14]. Combined therapy (*n* = 23) caused a significant decrease in circulating B cells, which was not observed with either anti-CTLA-4 (*n* = 8) or anti-PD-1 (*n* = 8) monotherapy. Combination therapy also increased CD21^lo^ B-cell subsets and plasmablasts, but B-cell changes did not correlate with clinical response. A strong correlation between early B-cell changes and the risk of subsequent immune-related AEs was observed, highlighting that B-cell monitoring might identify patients that could be at risk for autoimmune toxicity [14].

MDSCs may also play a role in cancer progression and may be an important biomarker for monitoring clinical outcome and response to therapy. Some studies in patients with metastatic melanoma treated with ipilimumab have indicated that blood levels of MDSCs inversely correlate with OS [15, 16], and a reduction in circulating MDSCs in local or regionally advanced metastatic melanoma after neoadjuvant ipilimumab treatment correlated with improved PFS [17]. Nonetheless, the specificity for cancer is not clear because MDSCs can expand in non-cancerous settings [46]. In addition, bona-fide markers for accurate characterization of different MDSC subsets in humans are not well standardized.

### Other cancers

In one of the initial attempts to profile circulating immune cells in early-stage NSCLC patients treated with neoadjuvant chemotherapy and ipilimumab [19], although chemotherapy had little effect on circulating immune cells, ipilimumab activated both CD4^+^ and CD8^+^ lymphocytes. In particular, CD4^+^ cells had increased surface expression of inducible co-stimulator (ICOS), HLA-DR, CTLA-4, and PD-1. In addition, tumor-infiltrating lymphocytes contained highly activated CD4^+^ and CD8^+^ T cells, indicating the tumors provided an immunogenic environment [19].

In a study of six patients with localized bladder cancer, those treated with ipilimumab had increased expression of ICOS on their CD4 T cells, both in the peripheral blood and the tumor [20]. The CD4^+^ICOS^hi^ T cells from treated patients produced more interferon-gamma (IFN-γ) than those from healthy donors or untreated patients [20], and the increase in CD4^+^ICOS^hi^ T cells associated with an increase in the ratio of effector cells to Tregs. A similar result was reported in a phase I study of 26 patients with advanced breast cancer treated with tremelimumab and exemestane [21]. However, this combination regimen showed limited clinical activity and was not developed further.

RNA analyses and flow cytometry of PBMCs found the extent of expression of inflammatory transcripts in the tumor and the percentages of circulating central memory (CM) and effector CD4^+^ and CD8^+^ T cells correlated in a study of patients with melanoma (*n* = 43) and non-squamous NSCLC (*n* = 40), expressed as independent CD4^+^ and CD8^+^ CM/effector T-cell ratios [22]. High CM/effector T-cell ratios correlated with inflamed tumors. As tumor T-cell infiltration is generally associated with favorable responses to checkpoint inhibitors, it was tested whether high CM/effector T-cell ratios at baseline correlated with clinical outcome in 22 patients with NSCLC treated with nivolumab [22]. In this cohort, patients with high CM/effector T-cell ratios experienced extended PFS compared with patients with low ratios [22]. In a study of patients with NSCLC (*n =* 29) receiving PD-1 targeted therapies, early on-treatment increases in PD-1^+^CD8^+^ T cells associated with clinical response [23]. No patients presenting late PD-1^+^CD8^+^ T-cell responses achieved partial clinical responses (≥6 weeks from treatment initiation) [23]. Hence, monitoring selected T-cell subsets before or during treatment in NSCLC may yield informative data on outcomes, although these findings require confirmation in larger studies.

### CD3 bispecific antibodies

The retargeting of T cells or other effector cells to tumors can be achieved using bispecific antibodies that simultaneously bind to target tumor cells and target effector cells [47]. The bispecific antibody catumaxomab (anti-EpCAM/anti-CD3; binds Fc-γ receptors on accessory immune cells) was the first bispecific approved by the European Medicines Agency for the treatment of malignant ascites. In a phase II/III trial of 258 patients with malignant ascites, catumaxomab with paracentesis showed clinical benefit vs paracentesis alone [48]. In a separate post hoc analysis of the same phase II/III trial, the relative lymphocyte count in peripheral blood before therapy predicted catumaxomab benefit. In patients with relative lymphocyte count > 13%, favorable OS was associated with catumaxomab treatment, with a mean OS benefit of 131 days and a 6-month survival rate of 37.0%, compared with 5.2% for paracentesis alone [25].

Tregs may also play a role in tumor development and immunosuppression by down-regulating effector cells. In a study of 42 patients with relapsed/refractory acute lymphoblastic leukemia (ALL) administered blinatumomab, a bispecific T-cell engager antibody directed against CD19 and CD3 antigens, a high percentage of peripheral blood Tregs was observed in 20 unresponsive patients [26]. In treatment-insensitive samples, the active depletion of Tregs (by magnetic-bead separation) restored blinatumomab-triggered T-cell proliferation in vitro. It is possible that blinatumomab-activated Tregs mediated resistance, leading to IL-10 production, suppressed T-cell proliferation, and decreased CD8-mediated lysis of ALL cells [26].

Some reports have associated the accumulation of CD4^+^FOXP3^+^CD25^hi^ Tregs with poor prognosis owing to the suppression of anti-tumor immune response [49–56], and altered Treg number and function has been reported in patients receiving conventional or immune therapies [57–59]. It will be important to further characterize Tregs with novel markers in peripheral blood to examine their association with clinical response to immunotherapy.

### Cancer vaccines

Peptide-based vaccines cause specific T-cell responses against antigens selectively expressed by tumor cells, but only a subset of patients show a clinical response. In fact, this lack of significant clinical response vs standard-of-care therapies may have hindered the identification of strongly predictive biomarkers; this topic was recently comprehensively reviewed by van der Burg [60]. Interestingly, pre-existing immune reactivity to vaccine peptides has not consistently been a strong response predictor, likely related to T-cell exhaustion or other inhibitory factors. However, some peripheral immune changes of significance have been identified in several studies.

The use of ELISPOT assays has been effective in the analysis of the function of circulating antigen-specific T cells following vaccination. The ELISPOT assay allows for classification of antigen-specific cells in a platform that is easily adjusted for several secreted molecules or cell types. Following vaccination with melanoma peptides in a phase II trial of patients with metastatic melanoma, IFN-γ production by CD8^+^ T cells after ex vivo stimulation with the vaccinating melanoma peptides (measured by ELISPOT), but not the frequency or phenotype of antigen-specific T cells, correlated with clinical responses to the vaccine [27]. In a separate study of 43 patients with metastatic castration-resistant prostate cancer (mCRPC), a dendritic cell-based vaccine was combined with docetaxel treatment and compared with docetaxel monotherapy [28]. Prostate-specific antigen responses, measured by IFN-γ ELISPOT, were similar in patients treated with docetaxel alone and in combination therapy, and an on-treatment decline in MDSCs independently predicted disease-specific survival [28]. To identify possible predictive immune biomarkers, another study using ELISPOT sought to investigate if antigen-specific or antigen non-specific immunity measures before treatment with a DNA vaccine encoding prostatic acid phosphatase (PAP) were associated with a subsequent immune response [29]. Immune responders were defined as subjects who had PAP-specific IFN-γ release detected by ELISPOT. The presence and type of pre-existing regulatory-type antigen-specific T-cell immunity was most associated with the development of persistent IFNγ-secreting antigen-specific T cell immunity. Non-immune responder patients tended to have higher antigen-specific IL-10 secretion prior to vaccination (measured by enzyme-linked immunosorbent assay [ELISA]), warranting further study of IL-10 as a negative predictive biomarker for immune response to this DNA vaccine [29].

A trial of personalized peptide vaccination characterized the gene-expression profiles in peripheral blood of vaccinated patients with mCRPC, to elucidate prognostic biomarkers [30]. The analysis of pre-vaccination PBMCs by microarray found a number of genes differentially expressed between short-term (*n* = 20) and long-term (*n* = 20) survivors [30]. Using stepwise discriminant analysis to choose a gene set from differentially expressed genes in pre-vaccination PBMCs, short-term survivors were predicted with 80% accuracy by a combination of four genes: *LRRN3*, *PCDH17*, *HIST1H4C*, and *PGLYRP1*. This four-gene classifier was validated in an external cohort, with prognosis correctly predicted in 12 of 13 cancer patients [30]. The study also reported that pre-vaccination IL-6 levels were significantly elevated in short-term vs long-term survivors.

In a trial of ipilimumab with PSA-TRICOM vaccine in 30 patients with mCRPC, subsets of T cells, Tregs, NK cells, and MDSCs were phenotyped by flow cytometry. Lower baseline PD-1^+^Tim-3^NEG^ CD4 effector memory cells, and higher baseline PD-1^NEG^Tim-3^+^ CD8 and CTLA-4^NEG^ Tregs was associated with improved OS. An increase in Tim-3^+^ NK cells post- vs pre-vaccination was also associated with longer OS [31]. In another study of mCRPC, 28 patients received intradermal prostate GVAX vaccine and ipilimumab [32, 33]. Baseline elevated CD4^+^CTLA-4^+^ in peripheral blood predicted for survival, while high pre-treatment levels of CD14^+^HLA-DR-monocytic MDSCs associated with reduced OS. These findings across multiple studies hold promise for the identification of mCRPC patients who may benefit from vaccine therapy.

The TG4010 vaccine was tested in combination with chemotherapy vs chemotherapy alone in a phase IIb trial of 148 patients with NSCLC [34]. When lymphocytes were analyzed at baseline (in 138 patients with evaluable samples), the percentage of CD16^+^CD56^+^CD69^+^ cells, a phenotype of activated NK cells, was a potential predictor of outcome in patients receiving TG4010. Patients with a normal percentage of CD16^+^CD56^+^CD69^+^ lymphocytes at baseline (*n* = 101) who received TG4010 plus chemotherapy had a better clinical outcome compared with patients receiving chemotherapy alone (*n* = 37). In patients with a high percentage of CD16^+^CD56^+^CD69^+^ lymphocytes before treatment, those given TG4010 plus chemotherapy (*n* = 21) had a worse outcome than those given chemotherapy alone (*n* = 16) [34].

The mRNA-based therapeutic vaccine RNActive® CV9201 was tested in a phase I/IIa trial of patients with NSCLC, and changes in peripheral blood during the vaccination period were assessed to identify biomarkers correlating with clinical outcome [35]. Whole-genome expression profiling in a subgroup of 22 Stage-IV patients before and after treatment initiation was performed and analyzed using an approach based on blood transcriptional modules. Patients segregated into two main groups according to their transcriptional changes: one group had an upregulated expression signature associated with myeloid cells and inflammation; the other had enrichment in T cells and NK cells. Compared with baseline, patients with enriched T- and NK-cell modules exhibited significantly longer PFS and OS compared with patients with upregulated myeloid-cell and inflammatory modules. The findings were validated with separate flow-cytometry analyses [35].

Novel biomarkers were explored before treatment or during vaccination with three HLA-A*2402-restricted peptides in a vaccine study of patients with pancreatic cancer [36]. Peripheral blood samples were taken from 36 patients in a HLA-A*2402-matched group and 27 patients in a HLA-A*2402-unmatched group. High expression levels of PD-1 on CD4^+^ T cells negatively predicted OS in the HLA-A*2402-matched group, and the induction of cytotoxic T lymphocytes. Following treatment, poor outcome was significantly associated with upregulation of PD-1 and Tim-3 expression on CD4^+^ and CD8^+^ T cells in the matched group only [36].

The tumor antigen MUC1 is expressed in certain types of cancer [61, 62] and is strongly immunogenic [63–66]. In a recent study, predictive biomarkers for clinical responses to the MUC1-targeted dendritic cell-based vaccine were assessed in 40 patients with refractory NSCLC [37]. Patients with immune-related AEs (e.g. fever and skin reactions at the vaccination site) showed significantly longer survival times compared with patients who did not experience such reactions. Patients whose baseline peripheral white blood cells contained > 20.0% lymphocytes also experienced longer survival times [37].

### CAR T-cell therapy

Chimeric antigen receptor (CAR) T cells represent a major approach in cancer immunotherapy, demonstrating success in some patients with hematologic malignancies. CAR T cells are T cells collected from blood of patients with disease (autologous) or healthy donors (allogenic) and engineered to express synthetic receptors to target antigens. They are infused to target and destroy cancerous cells, while continuing to multiply in situ. In a study of CAR T-cell (tisagenlecleucel) therapy in 41 patients with chronic lymphocytic leukemia (CLL), sustained remission was seen in patients with increased CD27^+^CD45RO^−^CD8^+^ T cells, with memory-like characteristics, measured in blood by flow cytometry prior to CAR T-cell infusion [38]. A mechanistically relevant population of CD27^+^PD-1^−^CD8^+^ CAR T cells expressing high levels of the IL-6 receptor predicted response to therapy and tumor control. The authors suggested the effectiveness of CAR T-cell therapy for CLL may be enhanced by treatment with cellular products enriched in CD27^+^PD-1^−^CD8^+^ cells [38].

In a study of CAR T cells targeting CD19 in 15 patients with B-cell lymphoma or leukemia, immune status was important for response [39]. Peripheral blood was profiled using polymerase chain reaction, flow cytometry, and proteomic array. The best predictor of response involved high levels of IL-12, dendritic cell lysosome-associated membrane glycoprotein, Fas ligand and TNF-related apoptosis-inducing ligand, and a low proportion of monocyte-like MDSCs. High-baseline or increasing on-treatment MDSCs, and high IL-6, IL-8, NAP-3, PD-L1, and PD-L2 correlated with poorer survival [39].

CAR T-cell expansion correlated with objective response in a study of 101 patients with large B-cell lymphoma treated with axicabtagene ciloleucel, an autologous anti-CD19 CAR T-cell therapy [40]. The expansion was significantly associated with response, with an area under the curve within the first 28 days that was 5.4-times higher in responders vs non-responders [40]. Elevated serum IL-6, IL-10, IL-15, and IL-2Rα levels were associated with neurological events and cytokine-release syndrome, and could provide useful safety markers [40]. Interestingly, a lack of CAR T-cell persistence observed in a study of patients with B-cell ALL was associated with relapse [41]. CD19 CAR T cells manufactured from defined CD4^+^ and CD8^+^ T-cell subsets were given to 30 participants with blood collected pre- and post-infusion. The development of CD8^+^ immunity to the CAR product resulted in relapse associated with loss of CAR T cells [41]. Hence, on-treatment assessment of CAR T-cell persistence and expansion may guide decisions on patient intervention after treatment initiation.

In a study of six patients with colorectal cancer, in order to assess the potential anti-tumor activity of CAR T hepatic artery infusions for unresectable carcinoembryonic antigen (CEA)-positive liver metastases, CEA levels were used as a surrogate of anti-tumor activity [42]. Patients with a favorable CEA response to CAR T were significantly more likely to have had lower-fold changes in their neutrophil-to-lymphocyte ratio (NLR) vs patients who did not have a favorable CEA response [42]. The correlation between NLR variations and CEA levels suggests that NLR variations may be a useful surrogate marker of tumor response.

Another study assessed pre-infusion CAR product T-cell polyfunctionality, identifying a significant association between a pre-specified T-cell polyfunctionality strength index and clinical response [43]. The strength of polyfunctionality combined with either CAR T-cell expansion, or with baseline IL-15 serum levels significantly associated with clinical outcome, compared with either measure alone. Associations with clinical outcomes were stronger with polyfunctional CD4^+^ T cells compared with CD8^+^ T cells, and anti-tumor efficacy associated with polyfunctional IL-17A-producing T cells [43].

### Emerging peripheral immune assessments

Various reports have shown the utility of soluble factors including TGF-β1, IL-6, IL-8, and IL-10, as either predictive or prognostic factors for response to immunotherapy [18, 67–69]. For example, serum baseline IL-8 levels reflected and predicted response to anti-PD-1 treatment in patients with melanoma and NSCLC [18], while baseline IL-10 correlated with tumor relapse in melanoma [68]. The measurement of such cytokines can be readily assessed by ELISA, offering an easily automated, highly sensitive, accurate and straightforward approach to analyzing multiple samples simultaneously.

Neoantigen-specific T cells are considered important immunotherapy effectors, but isolating this rare cell population has proven challenging. A recent report presented a sensitive approach to detect these cells using neoantigens and fluorescent DNA barcodes, presented on nanoparticle scaffolds, which allowed multiplex capture and analysis in blood or tumor. The study found a correlation between the kinetics of tumor shrinkage and the abundance kinetics of neoantigen-specific T cells in PBMCs in a patient with melanoma responding to immunotherapy [70].

T-cell diversity is recognized as potentially important in the development of tumor responses and toxicities in patients receiving therapies such as checkpoint inhibitors or cancer vaccines. A study reported in 2014 performed deep sequencing of the complementarity-determining region 3 (CDR3) of the T-cell receptor (TCR) variable-beta (V-beta) to assess changes in T-cell clonality and diversification in peripheral blood lymphocytes of 21 patients with melanoma treated with tremelimumab [71]. A 30% increase in unique productive sequences of TCR V-beta CDR3 was observed in 19 patients, whereas two patients showed a 30% decrease. The changes were significant both for Shannon index diversity (*p* = 0.04) and richness (*p* = 0.01) [71]. The expansion of the number of TCR V-beta CDR3 sequences reflects a larger T-cell diversity following treatment and may constitute a pharmacodynamic effect relating to modulation of the human immune system with CTLA-4 blockade [71].

The results of TCR sequencing of tumor samples has also predicted response to pembrolizumab treatment [1], with higher baseline TCR clonality in tumors from responding patients with melanoma observed in a recent study. In a subsequent trial in patients with breast cancer, combining ipilimumab with cryoablation, compared with ipilimumab alone, resulted in significant clonal expansion, with an increase in the amount of peripheral blood and intratumoral T-cell clones, supporting further study of the utility of TCR sequencing as a biomarker for T-cell response to therapy [72]. In a recent trial of the PD-L1-blocking antibody atezolizumab in patients with urothelial cancer, improved PFS and OS were more likely when peripheral TCR clonality was lower than the median at pre-treatment [73]. In patients with clinical benefit, there was also a significant expansion of tumor-associated TCR clones in peripheral blood at 3 weeks of treatment. Another study assessing the TCR repertoires in the peripheral blood of patients with metastatic pancreatic cancer treated with ipilimumab alone or in combination with a GVAX vaccine found that low pre-treatment clonality and a high number of post-treatment expanded clones were associated with longer survival in patients receiving ipilimumab, but not in those given nivolumab [74]. There were also significant enlargements in TCR repertoire in patients receiving ipilimumab, particularly when given in combination with GVAX [74]. These studies suggest peripheral blood TCR diversity or clonality could potentially serve as a biomarker for the prediction of clinical response to immunotherapy. One critical issue with TCR repertoire is that data generated by various vendors and laboratories may differ due to use of different primer sets and protocols. The Adaptive Immune Receptor Repertoire (AIRR) community of the antibody society aims to address issues involved with immune repertoire sequencing from sample collection to data processing, annotations, and reporting [75].

In addition to markers already mentioned, the epigenetic modulation of genes has also been implicated in tumorigenesis. Epigenetic silencing has been shown to lower the expression of HLA genes in certain cases, leading to impairments in T-cell-mediated immunity [76]. Developments in NGS-based epigenetic analyses are allowing rapid investigation of samples for determining the root of the abnormalities [77, 78]. In addition, the analysis of regulatory non-coding RNAs (small RNA-seq) may also help to identify mechanisms of tumor evasion [79, 80], and germline genetics is evolving as a potential predictor of checkpoint inhibitor response [81]. Lastly, a recent study of serum metabolites by liquid chromatography–mass spectrometry in patients with melanoma or renal cell carcinoma treated with nivolumab reported increased kynurenine and kynurenine/tryptophan ratios that were associated with poorer OS, highlighting metabolic adaptions reflected in serum as another emerging marker of immunotherapy response [82].

### Harmonization and standardization of assays/testing

Currently there are no validated FDA-approved circulatory immunologic biomarkers in the field of oncology, making comparisons between studies difficult because of assay variability, different platforms, and lack of reference standards. Various programs are underway to help steer efforts towards providing standardized biomarkers for uniform clinical application. The Partnership for Accelerating Cancer Therapies (PACT), for example, is a 5-year public–private research collaboration totaling US$220 million launched by the National Institutes of Health (NIH), the Foundation for the NIH (FNIH), and 12 leading pharmaceutical companies [83]. PACT is focusing on approaches to identify, develop, and validate biomarkers to advance new cancer immunotherapies. The partnership is managed by FNIH, with the FDA serving in an advisory role [83].

The National Clinical Trials Network (NCTN) has also established a laboratory network to serve National Cancer Institute (NCI)-sponsored clinical trials involving cancer immunotherapy. The Cancer Immune Monitoring and Analysis Centers (CIMAC) were created to perform biomarker assays for NCI-funded trials, providing consistent platforms, methodologies, and data-analysis approaches, furthering the harmonization of immuno-oncology biomarkers across the NCTN. Currently, harmonization and standardization of key platforms (including circulating cell-based analyses) is underway to ensure quality and consistent data across the different centers. In addition, as data accumulate over time, the associated Cancer Immunologic Data Commons will serve as a centralized data repository, providing access to high-quality data for the entire research community.

In 2016, Working Group 1 of the Society for Immunotherapy of Cancer Immune Biomarkers Task Force published their perspective on the pre-analytical and analytical, and the clinical and regulatory aspects of the validation process as applied to predictive biomarkers for cancer immunotherapy [84, 85]. For pre-analytical validation, they highlight the need to evaluate factors that may affect assay performance, such as sample-related variability, and discuss the importance of blood collection and storage media, citing best practice guidelines for biospecimen collection. Once an assay is established, the inclusion of appropriate control materials to ensure the assay is working accurately and reproducibly is also key. For a biomarker assay to be “fit-for-purpose”, the assay should clear a number of hurdles: 1) It must accurately and reliably measure the analyte in the population of interest; 2) Clinical validation must show the assay separates a population into two or more distinct groups with different biological characteristics or clinical outcomes; 3) For the assay to have clinical utility, its use must result in patient benefit or add value to patient management decision-making compared with current practices [84, 85].

### Future directions for clinical trials

As biomarkers and patient-enrichment strategies evolve, clinical trial designs also need to evolve. The NCI is in the early stages of developing a centralized screening protocol, called iMATCH, to prospectively identify patients for selection or stratification into immuno-oncology therapeutic trials. The specific biomarkers used to select patients are still being determined, but various approaches are being considered. Once the screening biomarkers are finalized, multiple clinical protocols will be developed under this central screening platform. New agents or novel combinatorial regimens will be tested across different tumor types and different clinical settings. Currently, most immuno-oncology trials do not employ upfront stratification or selection, and trial designs such as these may help to enrich for sensitive patient populations. Novel approaches to trial design such as this can be more efficient, especially when biomarker prevalence is low, and allows flexibility in adding and dropping treatment arms. However, these approaches may require a large number of drugs, well-defined biomarkers, and regulatory oversight over both the drugs and the biomarkers, emphasizing that biomarker assays are as important to the trial as drug development.

## Conclusions

The development of peripheral biomarkers for immunotherapy approaches is a clinically important and rapidly emerging field. A number of clinical studies using various assays and platforms to monitor peripheral immune status point to the utility of these biomarkers as potential predictive and prognostic readouts. To fully realize their predictive potential, it is likely that integrated analysis of peripheral immune-based biomarkers at the cell, genomic, or epigenetic level, with tumor and/or clinical response measures will be required. The utilization of high content data-generating technologies, including multicolor flow and mass cytometry, whole transcriptome sequencing, epigenetic analysis, and multianalyte serum immunoassays provides a deeper and broader view of the peripheral immune system and its relationship to the tumor-immune microenvironment. Extracting predictive signatures from these data must first be analyzed retrospectively, then prospectively in clinical trials with defined patient populations and endpoints. Of critical importance, much is still to be done to standardize assays and harmonize approaches, and work is currently underway to address these issues. Further research to validate such biomarkers as being reproducible, sensitive, and specific, as well as being clinically meaningful, will help strengthen their case to best identify the right immunotherapy approach for a given patient.

## Data Availability

Not applicable (review article).

## Abbreviations

AE: Adverse event
ALL: Acute lymphoblastic leukemia
CAR: Chimeric antigen receptor
CDR3: Complementarity-determining region 3
CEA: Carcinoembryonic antigen
CLL: Chronic lymphocytic leukemia
CM: Central memory (cell)
EM1: Effector-memory type 1 (cell)
FDA: Food and Drug Administration
FLT3LG: fms-related tyrosine kinase 3 ligand
FNIH: Foundation for the National Institutes of Health
ICOS: Inducible co-stimulator
IL: Interleukin
MDSCs: Myeloid-derived suppressor cells
NCI: National Cancer Institute
NCTN: National Clinical Trials Network
NGS: Next-generation sequencing
NIH: National Institutes of Health
NK: Natural killer (cell)
NLR: Neutrophil-to-lymphocyte ratio
NSCLC: Non-small cell lung carcinoma
OS: Overall survival
PACT: Partnership for Accelerating Cancer Therapies
PBMC: Peripheral blood mononuclear cell
PD-1: Programmed cell death protein 1
PD-L1/2: Programmed death ligand 1/2
PFS: Progression-free survival
RNA-seq: RNA sequencing
TCR: T-cell receptor
TEMRA: Terminally differentiated effector-memory T cells
T_ex_: Exhausted-phenotype cd8 t (cell)
TMB: Tumor mutational burden
Tmem: Memory t (cell)
Treg: Regulatory T (cell)
V-beta: Variable-beta
